# Impact of the addition of azithromycin to antimalarials used for seasonal malaria chemoprevention on antimicrobial resistance of *Streptococcus pneumoniae*

**DOI:** 10.1111/tmi.13321

**Published:** 2019-11-13

**Authors:** Soumeya Hema‐Ouangraoua, Abdoul Aziz Maiga, Matthew Cairns, Issaka Zongo, Nikiema Frédéric, Rakiswendé Serge Yerbanga, Boubou Tamboura, Henry Badji, Georgia Gore‐Langton, Irene Kuepfer, Halidou Tinto, Issaka Sagara, Alassane Dicko, Samba O. Sow, Daniel Chandrahoman, Brian Greenwood, Jean Bosco Ouedraogo

**Affiliations:** ^1^ Centre MURAZ Bobo‐Dioulasso Burkina Faso; ^2^ Institut de Recherche en Sciences de la Santé Bobo‐Dioulasso Burkina Faso; ^3^ Centre pour le Développement des Vaccins du Mali Bamako Mali; ^4^ London School of Hygiene & Tropical Medicine London UK; ^5^ Malaria Research and Training Centre (MRTC) University of Science, Techniques and Technologies of Bamako Bamako Mali

**Keywords:** pneumococcal carriage, azithromycin, resistance, Sub‐Saharan Africa, portage de pneumocoque, azithromycine, résistance, Afrique subsaharienne

## Abstract

**Objective:**

A trial was conducted in Burkina Faso and Mali to investigate whether addition of azithromycin to the antimalarials used for seasonal malaria chemoprevention reduces mortality and hospital admissions of children. We tested the sensitivity of nasal isolates of *Streptococcus pneumoniae* obtained during this trial to azithromycin and other antibiotics.

**Methods:**

Azithromycin or placebo was administered monthly, in combination with the antimalarials used for seasonal malaria chemoprevention, for four months, over the annual malaria transmission seasons of 2014, 2015, and 2016. Nasopharyngeal swabs were collected from 2773 Burkinabe and 2709 Malian children on seven occasions: in July and December each year prior to and after drug administration, and at a final survey in early 2018. Pneumococci were isolated from nasopharyngeal swabs and tested for sensitivity to azithromycin and other antibiotics.

**Results:**

A total of 5482 samples were collected. In Burkina Faso, the percentage of pneumococcal isolates resistant to azithromycin among children who had received it increased from 4.9% (95% CI: 2.4%, 9.9%) before the intervention to 25.6% (95% CI: 17.6%, 35.7%) afterward. In Mali, the increase was from 7.6% (95% CI: 3.8%, 14.4%) to 68.5% (95% CI: 55.1%, 79.4%). The percentage of resistant isolates remained elevated (17.7% (95% CI: 11.1%, 27.1%) in Burkina Faso and 19.1% (95% CI: 13.5%, 26.3%) in Mali) among children who had received azithromycin 1 year after stopping the intervention. An increase in resistance to azithromycin was also observed in children who had received a placebo but it was less marked.

**Conclusion:**

Addition of azithromycin to the antimalarial combination used for seasonal malaria chemoprevention was associated with an increase in resistance of pneumococci to azithromycin and erythromycin, which persisted 1 year after the last administration of azithromycin.

## Abstract (fr)

**Objectif:**

Un essai a été mené au Burkina Faso et au Mali pour investiguer si l’addition d’azithromycine aux antipaludéens utilisés dans le cadre de la chimioprévention du paludisme saisonnier réduisait la mortalité et les hospitalisations d’enfants. Nous avons testé la sensibilité à l'azithromycine et à d'autres antibiotiques pour les isolats nasaux de *Streptococcus pneumoniae* obtenus lors de cet essai.

**Méthodes:**

L'azithromycine ou un placebo a été administré mensuellement, en association avec les antipaludéens utilisés pour la chimioprévention du paludisme saisonnier, pendant 4 mois, durant les saisons de transmission annuelle du paludisme de 2014, 2015 et 2016. Des échantillons nasopharyngés ont été prélevés sur écouvillons chez 2.773 enfants burkinabés et 2.709 enfants maliens lors de 7 occasions: en juillet et en décembre chaque année avant et après l'administration du médicament, ainsi que lors d'une surveillance finale au début de 2018. Les pneumocoques ont été isolés à partir d'écouvillons nasopharyngés et soumis à des tests de sensibilité à l'azithromycine et à d'autres antibiotiques.

**Résultats:**

5.482 échantillons ont été collectés. Au Burkina Faso, le pourcentage d’isolats de pneumocoque résistants à l’azithromycine chez les enfants qui l’avaient reçu était passé de 4,9% (IC95%: 2,4%, 9,9%) avant l’intervention à 25,6% (IC95%: 17,6–35,7%) après. Au Mali, l'augmentation est passée de 7,6% (IC95%: 3,8–14,4%) à 68,5% (IC95%: 55,1–79,4%). Le pourcentage d'isolats résistants est resté élevé (17,7% (IC95%: 11,1–27,1%) au Burkina Faso et 19,1% (IC95%: 13,5–26,3%) au Mali) chez les enfants ayant reçu l'azithromycine un an après arrêter l'intervention. Une augmentation de la résistance à l'azithromycine a également été observée chez les enfants ayant reçu un placebo, mais elle était moins marquée.

**Conclusion:**

L’ajout d'azithromycine à la combinaison antipaludique utilisée pour la chimioprévention du paludisme saisonnier était associé à une augmentation de la résistance du pneumocoque à l'azithromycine et à l'érythromycine, qui persistait un an après la dernière administration d'azithromycine.

## Introduction

In Burkina Faso and Mali, malaria continues to be a burden with a large number of cases and high mortality rates despite control efforts. In 2016, WHO estimated 7.9 million malaria cases with 21 300 fatalities in Burkina Faso and 7.2 million cases with 12 400 fatalities in Mali 1. Malaria is highly seasonal; 60% to 80% of cases occur during the raining season in both countries.

Mass drug administration (MDA) with azithromycin (AZ) is being widely deployed as a highly effective method for the control of trachoma 2. The incidence of respiratory, gastrointestinal and skin infections, and malaria 3, 4, 5, 6, 7, 8 is lower in children who participated in mass AZ campaigns. An additional, surprising finding was detection of a more than 50% reduction in mortality in children who participated in an AZ MDA program in Ethiopia, a reduction that was sustained over a period of 26 months 9, 10. This unexpected finding led to the MORDOR (Macrolides Oraux pour Réduire les Décès avec un Oeil sur la Résistance) trial, which investigated the impact of two rounds of AZ MDA on mortality in children under the age of 5 years in Malawi, Niger, and Tanzania 11. An overall reduction in mortality of 13.5 % was observed, with the reduction being most marked in Niger and in children under the age of 1 year.

On the basis of the findings of the study in Ethiopia, we hypothesized that adding AZ to the antimalarial combination sulphadoxine‐pyrimethamine and amodiaquine (SP + AQ) used for seasonal malaria chemoprevention (SMC) would further reduce child mortality and severe morbidity, and we conducted a trial in 19 200 children aged 3–59 months in Burkina Faso and Mali to investigate this hypothesis. Modest reductions in the incidence of gastrointestinal, respiratory tract and skin infections, and non‐malaria fevers were seen, but the addition of AZ to the antimalarial used for SMC had no impact on child mortality or hospital admissions 12.

A concern over the use of AZ for MDA programs is that this will enhance resistance to macrolide antibiotics. An increase in the resistance of *Streptococcus pneumoniae* to AZ has been observed in several MDA studies, although this has often been only short term 5, 13, 14, 15, 16, 17, 18, 19. An increase in resistance of *Staphylococcus aureus* to AZ after MDA for trachoma control has also been reported 20. For these reasons, the antibiotic sensitivity of nasopharyngeal isolates of *S. pneumoniae* was studied during the SMC + AZ trial and the results from this study are presented in this paper.

## Methods

### Design and conduct of the SMC + AZ trial

Details of the trial in Burkina Faso and Mali to investigate the impact of adding AZ to the antimalarial drugs used for SMC have been published previously 12. In brief, 19 200 children aged 3–59 months were randomised to receive SP and AQ with either AZ or placebo. Randomization was by household. Infants aged 3–11 months received SP 250 mg/12.5 mg and AQ 75 mg on day 1 and AQ 75 mg on days 2 and 3. In addition, they received AZ 100 mg or matching AZ placebo on days 1, 2, and 3. Children aged 1–4 years received double these doses. SP + AQ was supplied by Guilin Pharmaceutical (Shanghai, China), and AZ and matching placebo were supplied by CIPLA (Mumbai, India). All doses of treatments were given by trial staff. Coverage with the monthly treatments was high, with more than 80% of children receiving three or four rounds of treatment each year. Deaths, hospital admissions, and attendances at clinics were recorded throughout the study period as described earlier 12. Cross‐sectional surveys were undertaken at the end of each malaria transmission season. The overall outline of the study is shown in Figure S1.

### Nasopharyngeal carriage surveys

Nasopharyngeal swabs were collected from 2773 Burkinabe and 2709 Malian children at cross‐sectional surveys at seven time points. Children were randomly selected by an independent statistician, with a new sample drawn for each time point. Swabs were collected in July 2014, 2015, and 2016 just before the first round of AZ or placebo was given and in December of each of these years 4–6 weeks after the last round of AZ or placebo had been given (hereafter referred to as pre‐ and post‐2014 etc.). Swab samples were also taken early in 2018, 1 year after the last administration of AZ or placebo.

Randomized children were gathered in a health facility and trained staff took samples on‐site while completing the required documents. A sample was taken from the posterior wall of the child's nasopharynx using a calcium alginate swab (FLOQSwabs, Copan Diagnostics Inc., Murrieta, CA, USA) and immediately transferred to a cryotube containing skimmed milk‐tryptone‐glucose‐glycerol medium (STGG). The cryotubes were labeled and placed in a cold box prior to transfer to the laboratory within 8 h of collection and stored at −80 °C until analyzed 21, 22.

### Laboratory methods

Standard protocols for the analysis of nasopharyngeal samples were used 21; these are described in the supplement.

### Statistical analysis

The primary study endpoint was the prevalence of nasal carriage isolates of *S. pneumoniae* resistant to AZ at the seven time points described above. A secondary endpoint was the overall prevalence of pneumococcal carriage in the two intervention groups at the same time points. Exploratory endpoints included the analysis of the sensitivity of pneumococci to other antibiotics.

### Sample size

A sample size of 400 children per survey per country was chosen for the nasopharyngeal substudy on the basis that the pneumococcal carriage prevalence would be 50%, and thus, 200 samples would be positive and available for resistance assays at each survey in each country. Assuming that the prevalence of resistance was 50% among the 200 samples available for resistance assays, the precision of the estimate of resistance for each country, at each time point, would be within 15% of the true value.

### Trial oversight

The trial was approved by ethics committees of the London School of Hygiene & Tropical Medicine, of the Malaria Research and Training Centre, Bamako, and of the Ministry of Health in Ouagadougou. The trial was also approved by the national regulatory authorities in Burkina Faso and Mali, and registered on Clinicaltrials.gov (NCT02211729).

Written consent was obtained from parents or guardians for inclusion of a child in the overall trial, and further consent was obtained from parents or guardians of children selected for the pneumococcal carriage substudy. A data safety monitoring board reviewed serious adverse events and monitored the trial's overall progress. An international steering committee reviewed the protocol and provided advice throughout the course of the study.

## Results

### Study population and samples

A total of 5482 nasopharyngeal specimens were collected, 2773 from children in Houndé, Burkina Faso (1379 from the AZ and 1394 from the placebo group) and 2709 from children in Bougouni, Mali (1346 from the AZ and 1363 from the placebo group). Sex and age distribution of study children were well balanced between the AZ and placebo groups, and the prevalence of pneumococcal carriage was comparable in each group at baseline in both countries (Tables 1 and 2). The numbers of swabs obtained at each survey and tested for antibiotic resistance are shown in Tables 3 and 4.

**Table 1:** General characteristics of children in the two study groups (Burkina Faso)

Characteristic	Percentage (*n*) of children by survey and country													
	Pre‐2014 (*N* = 430)		Post‐2014 (*N* = 418)		Pre‐2015 (*N* = 401)		Post‐2015 (*N* = 388)		Pre‐2016 (*N* = 385)		Post‐2016 (*N* = 396)		2018 (*N* = 355)	
	SMC + P	SMC + AZ	SMC + P	SMC + AZ	SMC + P	SMC + AZ	SMC + P	SMC + AZ	SMC + P	SMC + AZ	SMC + P	SMC + AZ	SMC + P	SMC + AZ
Total children (*n*)	213	217	213	205	196	205	197	191	192	193	201	195	182	173
Male % (*n*)	53.1 (112)	47.5 (103)	50.2 (107)	47.3 (97)	53.1 (104)	55.1 (113)	50.3 (99)	45.0 (86)	54.2 (104)	54.9 (106)	50.75 (102)	53.9 (105)	52.8 (96)	53.8 (93)
Age														
<12 months	21.6 (46)	24.0 (52)	11.3 (24)	10.2 (21)	15.8 (31)	12.7 (26)	7.6 (15)	8.9 (17)	16.7 (32)	13.0 (25)	8.0 (16)	8.7 (17)	0.0 (0)	0.0 (0)
12–23 months	17.4 (37)	17.5 (38)	20.7 (44)	20.5 (42)	15.3 (30)	25.9 (53)	25.4 (50)	20.9 (40)	16.2 (31)	24.4 (47)	24.9 (50)	26.2 (51)	0.0 (0)	0.0 (0)
24–35 months	23.0 (49)	23.5 (51)	21.1 (45)	21.5 (44)	24.0 (47)	20.0 (41)	22.3 (44)	23.6 (45)	22.9 (44)	22.3 (43)	19.4 (39)	21.5 (42)	20.3 (37)	20.2 (35)
36–47 months	19.7 (42)	16.6 (36)	21.1 (45)	20.0 (41)	22.4 (44)	21.5 (44)	20.8 (41)	25.7 (49)	25.0 (48)	19.2 (37)	20.4 (41)	14.4 (28)	18.7 (34)	20.2 (35)
48 months+	18.3 (39)	18.4 (40)	25.8 (55)	27.8 (57)	22.4 (44)	20.0 (41)	23.9 (47)	20.9 (40)	19.3 (37)	21.2 (41)	27.4 (55)	29.2 (57)	61.0 (111)*	59.5 (103)*
PCV vaccination % (*n*)	NA	NA	NA	NA	64.2 (43)	52.3 (34)	57.9 (33)	71.2 (42)	95.2 (99)	89.4 (93)	87.0 (87)	87.0 (80)	NA	NA
*n* missing	NA	NA	NA	NA	129	140	140	132	88	89	101	103	NA	NA
Recent antibiotic use % (*n*)	NA	NA	6.10 (13)	5.85 (12)	8.67 (17)	14.6 (30)	6.60 (13)	6.28 (12)	6.77 (13)	6.74 (13)	4.48 (9)	4.10 (8)	NA	NA
Number with recent illness	NA	NA	20	18	43	50	19	19	32	38	16	12	NA	NA
Antibiotic use if recent illness % (*n*)	NA	NA	65.0 (13)	66.7 (12)	39.5 (17)	60.0 (30)	68.4 (13)	63.2 (12)	40.6 (13)	34.2 (13)	56.3 (9)	66.7 (8)	NA	NA
Pneumococcal carriage % (*n*)	69.0 (147)	65.9 (143)	69.5 (148)	55.1 (113)	57.7 (113)	50.2 (103)	72.6 (143)	64.9 (124)	53.7 (103)	49.7 (96)	49.3 (99)	44.1 (86)	64.3 (117)	55.5 (96)
Prevalence Ratio (95% CI)	0.95 (0.84, 1.09)		0.79 (0.68, 0.92)		0.87 (0.73, 1.05)		0.89 (0.78, 1.02)		0.93 (0.76, 1.12)		0.90 (0.72, 1.11)		0.86 (0.73, 1.03)	
*P*‐value	0.498		0.003		0.141		0.104		0.442		0.311		0.095	

SMC + AZ, seasonal malaria chemoprevention with azithromycin; SMC + P, seasonal malaria chemoprevention with placebo; PCV, pneumococcal conjugate vaccine.

* In 2018 survey, the age category ‘48 months+’ includes children up to 6 years of age due to this survey being conducted 1 year after administration of the last SMC round.

**Table 2:** General characteristics of children in the two study groups (Mali)

Characteristic	Percentage (*n*) of children by survey and country													
	Pre‐2014 (*N* = 342)		Post‐2014 (*N* = 407)		Pre‐2015 (*N* = 399)		Post‐2015 (*N* = 395)		Pre‐2016 (*N* = 381)		Post‐2016 (*N* = 385)		2018 (*N* = 400)	
	SMC + P	SMC + AZ	SMC + P	SMC + AZ	SMC + P	SMC + AZ	SMC + P	SMC + AZ	SMC + P	SMC + AZ	SMC + P	SMC + AZ	SMC + P	SMC + AZ
Total children (*n*)	174	168	203	204	205	194	197	198	190	191	187	198	207	193
Male % (*n*)	53.5 (93)	54.8 (92)	50.3 (102)	51.0 (104)	50.2 (103)	48.5 (94)	45.2 (89)	57.1 (113)	56.3 (107)	50.3 (96)	45.5 (85)	60.1 (119)	47.3 (98)	59.1 (114)
Age														
<12 months	13.8 (24)	11.3 (19)	10.8 (22)	11.8 (24)	11.7 (24)	12.9 (25)	7.6 (15)	11.6 (23)	13.7 (26)	12.6 (24)	9.6 (18)	9.1 (18)	0 (0.0)	0 (0.0)
12–23 months	18.4 (32)	22.6 (38)	21.2 (43)	20.6 (42)	20.5 (42)	24.2 (47)	18.3 (36)	16.2 (32)	17.4 (33)	25.7 (49)	19.8 (37)	21.2 (42)	5.3 (11)	5.7 (11)
24–35 months	21.3 (37)	19.6 (33)	22.2 (45)	21.1 (43)	19.5 (40)	20.6 (40)	23.4 (46)	24.2 (48)	24.7 (47)	17.3 (33)	23.0 (43)	23.7 (47)	22.7 (47)	21.2 (41)
36–47 months	27.0 (47)	23.2 (39)	23.6 (48)	23.0 (47)	24.9 (51)	19.6 (38)	22.3 (44)	21.7 (43)	24.2 (46)	22.0 (42)	21.9 (41)	21.7 (43)	21.7 (45)	21.2 (41)
48 months+	19.5 (34)	23.2 (39)	22.2 (45)	23.5 (48)	23.4 (48)	22.7 (44)	28.4 (56)	26.3 (52)	20.0 (38)	22.5 (43)	25.7 (48)	24.2 (48)	50.2 (104)*	51.8 (100)*
PCV vaccination % (*n*)	NA	NA	NA	NA	95.0 (151)	92.8 (129)	91.5 (139)	95.6 (153)	93.8 (151)	97.0 (164)	95.7 (154)	95.8 (158)	NA	NA
*n* missing	NA	NA	NA	NA	46	55	45	38	29	22	26	33	NA	NA
Recent antibiotic use % (*n*)	NA	NA	9.85 (20)	6.86 (14)	9.76 (20)	11.9 (23)	9.64 (19)	8.08 (16)	7.37 (14)	7.85 (15)	10.2 (19)	10.1 (20)	NA	NA
Number with recent illness	NA	NA	30	25	42	47	31	36	60	54	33	36	NA	NA
Antibiotic use if recent illness % (*n*)	0.0 (0)	0.0 (0)	66.7 (20)	56.0 (14)	47.6 (50)	48.9 (23)	61.3 (19)	44.4 (16)	23.3 (14)	27.8 (15)	57.6 (19)	55.6 (20)	NA	NA
Pneumococcal carriage % (*n*)	63.2 (110)	63.7 (107)	56.2 (114)	58.8 (120)	53.7 (110)	56.7 (110)	72.6 (143)	67.7 (134)	25.8 (49)	28.3 (54)	50.3 (94)	31.0 (61)	90.8 (188)	85.5 (165)
Prevalence Ratio (95% CI)	1.01 (0.85, 1.19)		1.05 (0.89, 1.24)		1.06 (0.88, 1.27)		0.93 (0.82, 1.06)		1.10 (0.79, 1.53)		0.62 (0.47, 0.80)		0.94 (0.88, 1.01)	
*P*‐value	0.929		0.588		0.550		0.282		0.586		<0.001		0.096	

SMC + AZ, seasonal malaria chemoprevention with azithromycin; SMC + P, seasonal malaria chemoprevention with placebo; PCV = pneumococcal conjugate vaccine.

* In 2018 survey, the age category ‘48 months+’ includes children up to 6 years of age due to this survey being conducted 1 year after administration of the last SMC round.

**Table 3:** The prevalence of azithromycin (AZ) and erythromycin (ERY) resistance in nasopharyngeal isolates of *Streptococcus pneumoniae* in Burkina Faso

	Survey	SMC + Placebo				SMC + Azithromycin				Prevalence Ratio (95% CI)	*P*‐value
		Carriage (*n*)	Tested (*n*)	Resistant (*n*)	Prevalence (%)	Carriage(*n*)	Tested (*n*)	Resistant (*n*)	Prevalence (%)		
Azithromycin disk assay	Pre‐2014	147	147	3	2.04	143	143	7	4.90	2.40 (0.63, 9.13)	0.199
	Post‐2014	148	148	8	5.41	113	113	14	12.4	2.29 (0.98, 5.37)	0.056
	Pre‐2015	113	113	5	4.42	103	103	8	7.77	1.76 (0.59, 5.21)	0.311
	Post‐2015	143	143	19	13.3	124	124	25	20.2	1.52 (0.88, 2.62)	0.134
	Pre‐2016	103	103	11	10.7	96	96	9	9.38	0.88 (0.38, 2.03)	0.761
	Post‐2016	99	99	13	13.1	86	86	22	25.6	1.95 (1.05, 3.61)	0.034
	2018	117	117	19	16.2	96	96	17	17.7	1.09 (0.59, 2.03)	0.785
Azithromycin E‐test	Pre‐2014	147	147	4	2.72	143	143	5	3.50	1.28 (0.35, 4.71)	0.705
	Post‐2014	148	148	8	5.41	113	113	15	13.3	2.46 (1.06, 5.68)	0.036
	Pre‐2015	113	113	7	6.19	103	103	12	11.7	1.88 (0.77, 4.61)	0.167
	Post‐2015	143	143	22	15.9	124	124	30	24.2	1.57 (0.96, 2.58)	0.073
	Pre‐2016*	103	12	11	91.7*	96	9	9	100*	NA	
	Post‐2016*	99	67	18	26.9*	86	67	30	44.8*	NA	
	2018	117	117	21	17.9	96	96	18	18.8	1.04 (0.58, 1.88)	0.884
Erythromycin disk assay	Pre‐2014	147	147	3	2.04	143	143	7	4.90	2.40 (0.63, 9.13)	0.199
	Post‐2014	148	148	8	5.41	113	113	14	12.4	2.29 (0.98, 5.37)	0.056
	Pre‐2015	113	113	5	4.42	103	103	8	7.77	1.76 (0.59, 5.21)	0.311
	Post‐2015	143	143	18	12.6	124	124	27	21.8	1.73 (1.00, 2.99)	0.049
	Pre‐2016	103	103	11	10.7	96	96	9	9.38	0.88 (0.38, 2.03)	0.761
	Post‐2016	99	99	13	13.1	86	86	21	24.4	1.86 (1.00, 3.47)	0.051
	2018	117	117	17	14.5	96	96	17	17.7	1.22 (0.64, 2.32)	0.548

* In 2016, the azithromycin E‐test was used only to confirm samples positive by the azithromycin disk assay, not to test all samples. Consequently, a prevalence ratio cannot be calculated, and the values in the ‘Prevalence’ column for the placebo and AZ groups should be interpreted as ‘the percentage of samples positive by disk assay that were also found to be positive by E‐test’.

**Table 4:** The prevalence of azithromycin (AZ) and erythromycin (ERY) resistance in nasopharyngeal isolates of *Streptococcus pneumoniae* in Mali

	Survey	SMC + Placebo				SMC + Azithromycin				Prevalence Ratio (95% CI)	*P*‐value
		Carriage (*n*)	Tested (*n*)	Resistant (*n*)	Prevalence (%)	Carriage(*n*)	Tested (*n*)	Resistant (*n*)	Prevalence (%)		
Azithromycin disk assay	Pre‐2014	110	109	4	3.67	107	106	8	7.55	2.06 (0.64, 6.62)	0.227
	Post‐2014	114	113	6	5.31	120	117	13	11.1	2.09 (0.81, 5.43)	0.129
	Pre‐2015	110	110	7	6.36	110	110	5	4.55	0.71 (0.23, 2.20)	0.558
	Post‐2015	143	143	4	2.80	134	134	10	7.46	2.67 (0.86, 8.27)	0.089
	Pre‐2016*	49	34	8	23.5*	54	40	8	20.0*	0.85 (0.36, 2.02)*	0.713
	Post‐2016*	94	81	25	30.9*	61	54	37	68.5*	2.22 (1.51, 3.27)*	<0.001
	2018	188	180	31	17.2*	165	152	29	19.1*	1.11(0.69, 1.77)*	0.669
Azithromycin E‐test	Pre‐2014†	110	0	0	0.00†	107	2	2	100†	NA	
	Post‐2014†	114	6	6	100†	120	13	13	100†	NA	
	Pre‐2015†	110	7	7	100†	110	5	5	100†	NA	
	Post‐2015†	143	4	4	100†	134	11	11	100†	NA	
	Pre‐2016†	49	8	5	62.5†	54	8	5	62.5†	NA	
	Post‐2016†	94	25	17	68.0†	61	36	27	75.0†	NA	
	2018	188	36	34	94.4†	165	29	29	100.0†	NA	
Erythromycin disk assay	Pre‐2014	110	109	4	3.67	107	106	8	7.55	2.06 (0.64, 6.62)	0.227
	Post‐2014	114	113	6	5.31	120	117	13	11.1	2.09 (0.81, 5.43)	0.129
	Pre‐2015	110	110	7	6.36	110	110	5	4.55	0.71 (0.23, 2.20)	0.558
	Post‐2015	143	143	4	2.80	134	134	12	8.96	3.20 (1.06, 9.65)	0.039
	Pre‐2016*	49	34	4	11.8*	54	40	9	22.5*	1.91 (0.64, 5.75)*	0.248
	Post‐2016*	94	81	23	28.4*	61	53	35	66.0*	2.33 (1.54, 3.51)*	<0.001
	2018	188	180	27	15.0*	165	152	25	16.4*	1.10 (0.66, 1.83)*	0.726

* In 2016 and 2018, not all positive samples were tested by disk assay; prevalences and prevalence ratios reflect the prevalence among those samples that were tested.

^†^ The azithromycin E‐test was used only to confirm samples positive by the azithromycin disk assay, not to test all samples. Consequently, a prevalence ratio cannot be calculated, and the values in the ‘Prevalence’ column for the placebo and AZ groups should be interpreted as ‘the percentage of samples positive by disk assay that were also found to be positive by E‐test’.

A history of consumption of antibiotics other than AZ in the 30 days prior to the collection of a swab was reported by 4.1–14.6% of children in Burkina Faso and by 6.9–11.9% of children in Mali in different surveys (Table 1). Antibiotic use among the subset of children with a recent morbidity episode was higher, often exceeding 50%. Amoxicillin was the most commonly prescribed antibiotic, accounting for 120 of the 140 (85.7%) reported antibiotic treatments of participants in Burkina Faso and for 112 of 180 (62.2%) treatments in Mali. Erythromycin (10.0% of antibiotic treatments), co‐trimoxazole (10.6%), and metronidazole (21.7%) were also commonly used in Mali.

Pneumococcal conjugate (PCV13) vaccination was introduced into the Expanded Programme of Immunization in 2013 in Burkina Faso and in 2011 in Mali. Vaccination cards were often not available at the time of the annual census of all children in the trial. Vaccine coverage with PCV13 in Burkina Faso among children whose vaccine status was known was 58.3% when first measured before the 2015 malaria transmission season, but improved substantially in 2016 (Table 1). In Mali, PCV vaccine coverage was >90% at all survey contacts.

### Prevalence of nasopharyngeal carriage of *Streptococcus pneumoniae*

**Figure 1:**
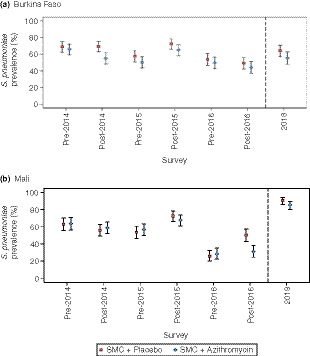
The prevalence of nasopharyngeal carriage of *Streptococcus pneumoniae* in three annual pre‐ and post‐intervention surveys and 1 year after the last post‐intervention survey. [Colour figure can be viewed at wileyonlinelibrary.com]

The overall prevalence of nasopharyngeal carriage of *S. pneumoniae* at baseline was 67.4% in Burkina Faso and 63.5% in Mali (Tables 1 and 2; Figure 1a,b). Overall carriage remained >50% at the five subsequent surveys during the study period, with the exception of the post‐2016 survey, when overall carriage was 46.7% in Burkina Faso and 40.4% in Mali. Carriage was generally lower in the AZ group in Burkina Faso but differences between the two study groups were not marked, with overlapping confidence intervals, apart from two occasions when prevalence was significantly lower in the AZ group: post‐2014 in Burkina Faso, with a prevalence ratio (PR) of 0.79 (0.68, 0.92; *P* = 0.003), and post‐2016 in Mali (PR 0.62 (0.47, 0.80), *P* < 0.001).

One year after the last administration of SMC with AZ, the prevalence of pneumococcal carriage was 60.0% in Burkina Faso (Table 1 and Figure 1a) and slightly lower in the group that had previously received AZ than in the placebo group (PR 0.86 (0.73, 1.03); *P* = 0.095). In Mali, the prevalence of pneumococcal carriage was 88.3% one year after the last administration of SMC with AZ (Table 2 and Figure 1b), again slightly but not markedly lower in the group that received AZ (PR 0.94 (0.88, 1.01); *P* = 0.096). Because of the unexpectedly high isolation rate in the last survey conducted in Mali, these samples were retested and a high isolation rate was confirmed (Table S1).

### *Resistance of pharyngeal isolates of* Streptococcus pneumonia *to azithromycin*

**Figure 2:**
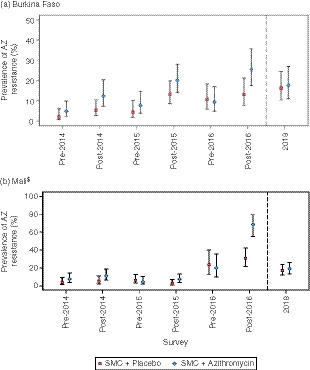
The prevalence of resistance to azithromycin among nasopharyngeal isolates of *Streptococcus. pneumoniae* in three annual pre‐ and post‐intervention surveys and 1 year after the last post‐intervention survey was done (a) Burkina Faso and (b) Mali*. Results from disk diffusion assays. *In Mali in 2016, not all positive samples were tested by disk assay; prevalences reflect the percentage positive among those samples that were tested. [Colour figure can be viewed at wileyonlinelibrary.com]

At baseline, the overall prevalence of resistance to AZ among nasopharyngeal isolates of *S. pneumoniae* assessed using a disk diffusion assay was low in each country (3.4% in Burkina Faso and 5.6% in Mali). In Burkina Faso, the prevalence of AZ resistance at baseline was 3.1% using the E‐test (Figure 2a,b). In Mali, the E‐test was used only to confirm resistance in positive samples identified by the disk diffusion assay and, at baseline, only two samples were tested, both of which were positive with each assay.

In Burkina Faso, results obtained using the disk diffusion assay showed that the prevalence of resistance to AZ increased over time in both study groups, reaching approximately 10% or more from the post‐2015 survey onwards. Prevalence was higher in the AZ group at each of the first six surveys, apart from the pre‐2016 survey, although differences were only marked at two time points: post‐2014 and post‐2016 (PR 2.29 (0.98, 5.37), (*P* = 0.056) and PR 1.95 (1.05, 3.61), (*P* = 0.034), respectively). One year after the last administration of AZ or placebo, the prevalence of resistance was markedly higher than the prevalence seen at baseline: 16.2% in the placebo group and 17.7% in the AZ group. Similar results were obtained with the AZ E‐test (Oxoid Ltd, Basingstoke, UK), although due to a shortage of test strips in 2016, not all samples positive for carriage could be tested at the pre‐2016 and post‐2016 surveys.

In Mali, the prevalence of AZ resistance according to the disk diffusion assay increased relative to baseline but remained below 10% in 2014 and 2015. The prevalence of AZ resistance exceeded 20% in both groups at both the pre‐2016 and post‐2016 surveys and was markedly higher in the AZ group at the post‐2016 survey: PR 2.22 (1.51, 3.27); *P* < 0.001). At the first four surveys (pre‐2014 to post‐2015), all samples positive by disk assay were confirmed positive by E‐test; approximately two‐thirds of samples positive by disk assay were confirmed to be positive by E‐test at the 2016 surveys. Because of the very high level of resistance to AZ found in the post‐2016 survey, these samples were retested and a high level of concordance between the two sets of testing was found (Table S2).

Analysis of changes in the pattern of resistance to AZ over time by age group did not show any age effect in either Burkina Faso or Mali (Figure S2).

### Resistance of pharyngeal isolates of *Streptococcus pneumoniae* to erythromycin

Resistance to erythromycin, assessed with a disk diffusion assay, showed a very similar pattern to that seen for AZ (Figures S3 and S4). In Burkina Faso, the prevalence of resistance increased from a prevalence at baseline of 3.5%, exceeding approximately 10% at all time points after the post‐2015 survey. The prevalence of resistance was higher in the AZ than in the placebo group at all but one survey, but there was only weak statistical evidence of a true difference on two occasions: PR 1.73 (1.00, 2.99; *P* = 0.049) at the post‐2015 survey, and PR 1.86 (1.00, 3.47; *P* = 0.051) at the post‐2016 survey. There was no evidence of a difference persisting between the groups at the 2018 survey.

In Mali, the results for erythromycin also closely mirrored those obtained for AZ: There was a relatively slow increase in the prevalence of resistance during the first 2 years of the study, but from 2016 onwards the overall prevalence of resistance exceeded 10%. Prevalence was higher in the AZ group at all but one time point, with evidence of a true difference between the study groups in the post‐2015 survey [PR 3.20 (1.06, 9.65; *P* = 0.039)] and strong evidence of a true difference at the post‐2016 survey [PR 2.33 (1.54, 3.51; *P* < 0.001)]. At the 2018 survey, the prevalence of erythromycin resistance had dropped below 17% in both study groups and there was no evidence of a difference between groups.

### Resistance of pharyngeal isolates of *Streptococcus pneumoniae* to penicillin

In Burkina Faso, the E‐test was used to determine sensitivity to penicillin. The overall prevalence of resistance at the first survey was 13.5%; during the remainder of the study period, this ranged between a low of 5.1% at the pre‐2015 survey and a high of 22.5% at the post‐2015 survey, with no evidence of any difference between the study groups (Table 5). At the 2018 survey, the overall prevalence was 25.8%, again with no difference between study groups [PR 1.09 (0.69, 1.73; *P* = 0.71)].

**Table 5:** The prevalence of resistance in nasopharyngeal isolates of *Streptococcus pneumoniae* to penicillin and oxacillin in Burkina Faso and Mali

	Survey	SMC + Placebo				SMC + Azithromycin				Prevalence Ratio (95% CI	*P*‐value
		Carriage (*n*)	Tested (*n*)	Resistant (*n*)	Prevalence (%)	Carriage (*n*)	Tested (*n*)	Resistant (*n*)	Prevalence (%)		
Burkina Faso											
Oxacillin disk assay	Pre‐2014	147	147	37	25.2	143	143	34	23.8	0.94 (0.63, 1.42)	0.783
	Post‐2014	148	148	38	25.7	113	113	18	15.9	0.62 (0.37, 1.03)	0.064
	Pre‐2015	113	113	44	38.9	103	103	35	34.0	0.87 (0.61, 1.24)	0.452
	Post‐2015	143	143	44	30.8	124	124	33	26.6	0.86 (0.59, 1.26)	0.454
	Pre‐2016	103	103	39	37.9	96	96	27	28.1	0.74 (0.50, 1.11)	0.150
	Post‐2016	99	99	33	33.3	86	86	30	34.9	1.05 (0.70, 1.57)	0.826
	2018	117	117	40	34.2	96	96	36	37.5	1.10 (0.76, 1.57)	0.616
Penicillin E‐test	Pre‐2014	147	147	19	12.9	143	143	20	14.0	1.08 (0.60, 1.94)	0.791
	Post‐2014	148	148	21	14.2	113	113	16	14.2	1.00 (0.54, 1.84)	0.995
	Pre‐2015	113	113	5	4.4	103	103	6	5.8	1.32 (0.41, 4.20)	0.642
	Post‐2015	143	143	33	23.1	124	124	27	21.8	0.94 (0.60, 1.48)	0.799
	Pre‐2016	103	103	17	16.5	96	96	19	19.8	1.20 (0.66, 2.17)	0.549
	Post‐2016	99	99	11	11.1	86	86	12	14.0	1.26 (0.58, 2.70)	0.559
	2018	117	117	29	24.8	96	96	26	27.1	1.09 (0.69, 1.73)	0.705
Mali											
Oxacillin disk assay	Pre‐2014	110	109	45	41.3	107	105	40	38.1	0.92 (0.66, 1.29)	0.639
	Post‐2014	114	113	17	15.0	120	117	18	15.4	1.02 (0.55, 1.91)	0.944
	Pre‐2015	110	110	21	19.1	110	110	18	16.4	0.86 (0.49, 1.51)	0.596
	Post‐2015	143	143	32	22.4	134	134	18	13.4	0.60 (0.35, 1.02)	0.057
	Pre‐2016	49	34	15	44.1	54	40	11	27.5	0.62 (0.33, 1.18)	0.145
	Post‐2016	94	80	32	40.0	61	53	23	43.4	1.08 (0.73, 1.62)	0.692
	2018	188	180	46	25.6	165	152	35	23.0	0.90 (0.61, 1.32)	0.593

### Resistance of pharyngeal isolates of *Streptococcus pneumoniae* to other antibiotics

Resistance to ceftriaxone, norfloxacin, and vancomycin was measured only in isolates obtained in Burkina Faso. Overall, a very low prevalence of resistance to ceftriaxone was found using either a disk diffusion method or an E‐test over the whole study period (0.61% and 0.31% respectively). Resistance to norfloxacin, tested with a disk diffusion test, was slightly more frequent and found in 85 samples (5.3% of those tested) over the study period, ranging from 11 positives (3.8% of those tested) at baseline to a low prevalence at the end of the study period, with only one positive at the post‐2016 survey and three positives at the 2018 survey. No resistance to vancomycin was found in any of the 1633 isolates tested.

## Discussion

This study evaluated the impact of AZ, combined with SMC, given once a month for 4 months (August to November) over a 3‐year period (2014–2016) on the resistance of *S. pneumoniae* to AZ. This was a more intense treatment schedule than that used in previous MDA studies employing AZ for control of trachoma 7, 8, 13, 14, 15, 16, 17, 18, 19, 20, 23. The two regions of Houndé in Burkina Faso and Bougouni in Mali had received the last distribution of AZ (Zithromax) for control of trachoma in 2007 and 2011, respectively, and this is, therefore, unlikely to have affected the results of this study 24.

The overall prevalence of carriage of *S. pneumoniae* declined modestly over time in children who had received either AZ or placebo with the exception of an unexpected increase in the 2018 survey in Mali; this increase was confirmed on retesting so is likely to be a true, but unexplained, finding. Carriage tended to be lower in the AZ group than in the placebo group, but apart from two time points (post‐2014 in Burkina Faso and post‐2016 in Mali), differences were not large and may have been due to chance.

In both Burkina Faso and Mali, resistance of pharyngeal isolates of *S. pneumoniae* to AZ and erythromycin increased substantially during the course of the study and this persisted for a year after the last drug administration. This contrasts with the findings in most other studies in which the prevalence of resistance has usually returned close to baseline at surveys some months after the last drug administration 16, 18. There was strong statistical evidence of a difference between the AZ and placebo groups only at the post‐2015 survey in Burkina Faso and at the post‐2016 survey in Mali. Although study children were randomized by household, rather than individually, there may have been sufficient mixing between young children in neighboring households to dilute differences between the intervention groups; a cluster‐randomized village trial might have found more marked differences between study groups. As expected, patterns of resistance to erythromycin matched those seen for AZ. A modest level of resistance to penicillin, as assessed by the E‐test, was found but resistance to other antibiotics tested was rare.

Incorporation of AZ into the SMC treatment regimen did not have any significant impact on deaths or hospital admission due to non‐traumatic causes but addition of AZ did reduced the incidence of visits to a health facility or community health worker due to an acute respiratory tract, gastrointestinal or skin infection, and of non‐malaria fever by about 20% 11. These gains will need to be balanced against the costs of adding AZ to the SMC regimen, currently being assessed, and against the risk of inducing widespread resistance of *S. pneumoniae*, and perhaps other bacterial pathogens, including gastrointestinal pathogens, to macrolide antibiotics. Erythromycin and (particularly) AZ are currently used infrequently for treatment of young children at government‐supported clinics in the study areas, but they may be prescribed more frequently in pharmacies and private clinics and their loss to the list of effective and affordable antibiotics would be a significant one.

This study has some weaknesses. Although efforts were made to standardize laboratory procedures in Burkina Faso and Mali, some differences in methods emerged, in part because of difficulties in obtaining reagents at the appropriate time. Hence, it was decided to undertake separate analyses in each country rather than merging the data. Nevertheless, very similar results were found in each country. In addition, data on coverage with pneumococcal conjugate vaccine were not recorded for each study participant, but the information that was available indicates that a high proportion had received at least one dose of this vaccine.

Policymakers are currently considering the potential of widespread deployment of AZ mass drug administration as an infant survival strategy in countries where infant mortality remains high. The results of this study suggest that the potential for inducing resistance to macrolide antibiotics in important pathogens will need to be taken into consideration when policy decisions are being made on the costs and benefits of this intervention.

## Supporting information


**Figure S1.** Schematic showing timing of surveys for pneumococcal sampling in relation to study interventions and the malaria transmission seasons.

**Figure S2.** Results of resistance to azithromycin by age obtained during three annual pre‐and post‐intervention surveys and 1 year after the last post‐intervention survey was done in Burkina Faso (a) and Mali (b).

**Figure S3.** Results of disc diffusion assays for testing for resistance to erythromycin and its comparison to resistance to azithromycin in isolates obtained during three annual pre‐and post‐intervention surveys and 1 year after the last post‐intervention survey was done in Burkina Faso.

**Figure S4. **Results of disc diffusion assays for testing for resistance to erythromycin and its comparison to resistance to azithromycin in isolates obtained during three annual pre‐and post‐intervention surveys and 1 year after the last post‐intervention survey was done in Mali.
