# Supplementary material for: Impact of the addition of azithromycin to antimalarials used for seasonal malaria chemoprevention on antimicrobial resistance of Streptococcus pneumoniae
Source: Trop Med Int Health. 2019 Nov 13;24(12):1442–54. doi: 10.1111/tmi.13321 (PMC7687265; doi:10.1111/tmi.13321)

Figure S1. Schematic showing timing of surveys for pneumococcal sampling in relation to study interventions and the malaria transmission seasons.

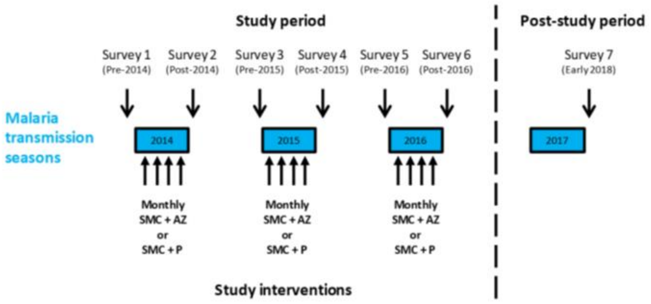

Supplement: Supplementary file 1 — Figure S1. Schematic showing timing of surveys for pneumococcal sampling in relation to study interventions and the malaria transmission seasons. [file TMI-24-1442-s001.pdf]
