# Supplementary figures and images for: Impact of the addition of azithromycin to antimalarials used for seasonal malaria chemoprevention on antimicrobial resistance of Streptococcus pneumoniae
Source: Trop Med Int Health. 2019 Nov 13;24(12):1442–54. doi: 10.1111/tmi.13321 (PMC7687265; doi:10.1111/tmi.13321)

a) Burkina Faso

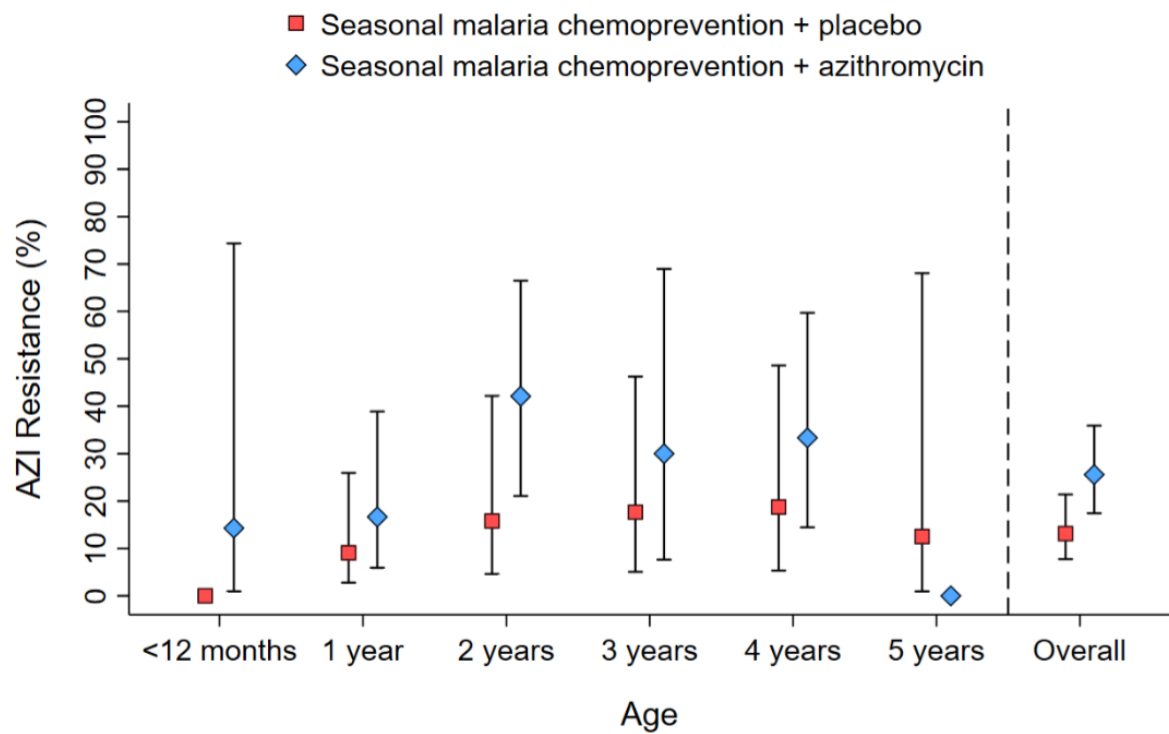

Disc Assay  
BF: post-2016 survey

b) Mali

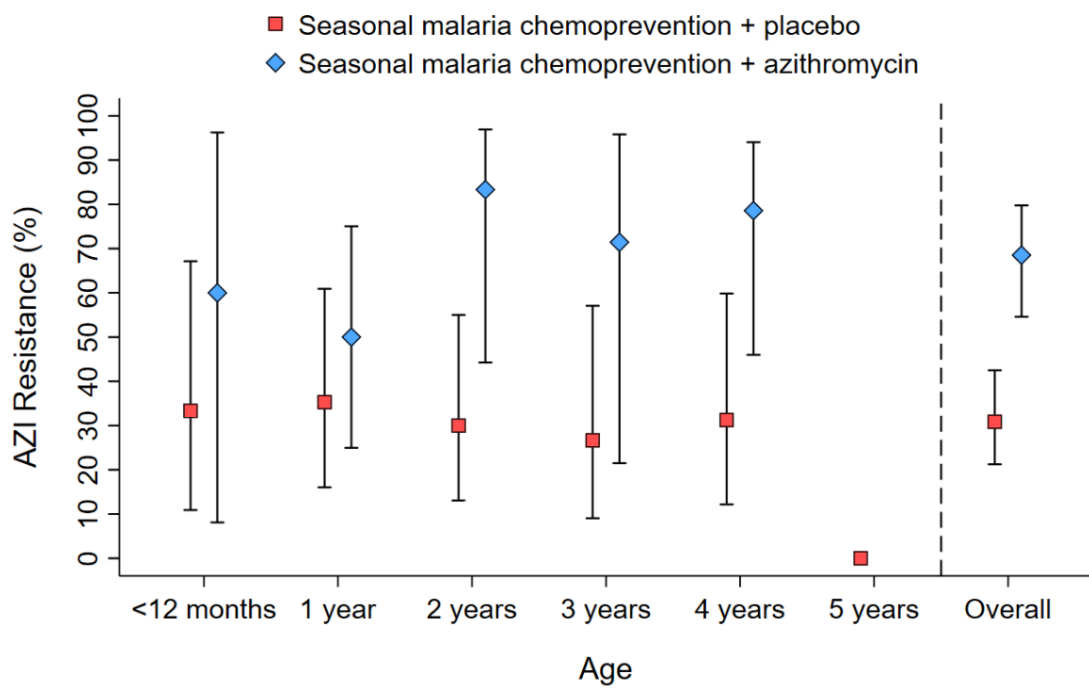

Disc Assay  
ML: post-2016 survey

Supplement: Supplementary file 2 — Figure S2. Results of resistance to azithromycin by age obtained during three annual pre‐and post‐intervention surveys and 1 year after the last post‐intervention survey was done in Burkina Faso (a) and Mali (b). [file TMI-24-1442-s002.pdf]
