# Supplementary material for: Impact of the addition of azithromycin to antimalarials used for seasonal malaria chemoprevention on antimicrobial resistance of Streptococcus pneumoniae
Source: Trop Med Int Health. 2019 Nov 13;24(12):1442–54. doi: 10.1111/tmi.13321 (PMC7687265; doi:10.1111/tmi.13321)
Supplement: Supplementary file 3 — Figure S3. Results of disc diffusion assays for testing for resistance to erythromycin and its comparison to resistance to azithromycin in isolates obtained during three annual pre‐and post‐intervention surveys and 1 year after the last post‐intervention survey was done in Burkina Faso. [file TMI-24-1442-s003.pdf]

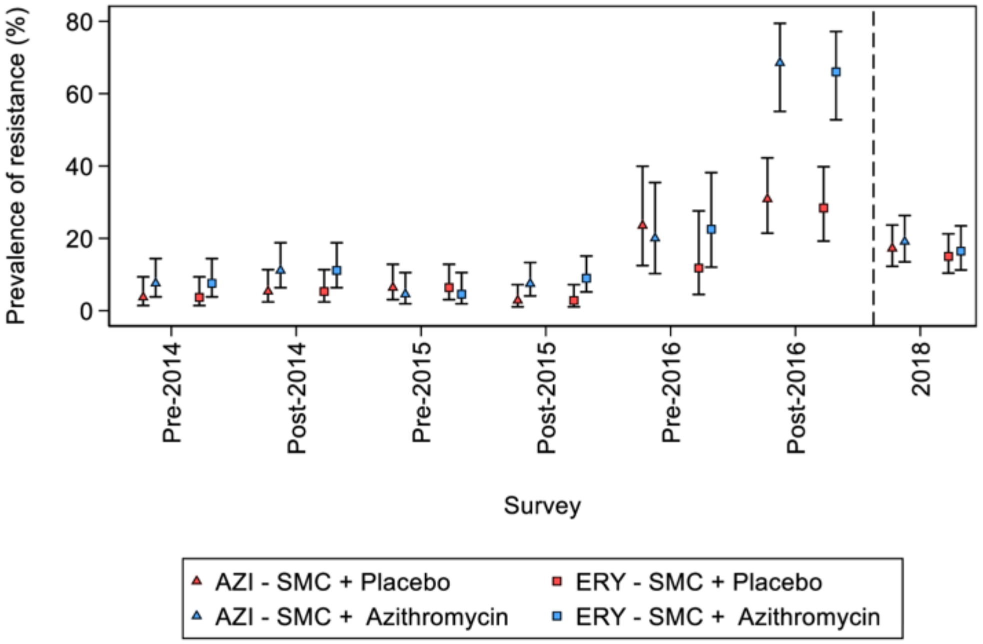

$ In Mali in 2016, not all positive samples were tested by disc assay; prevalence reflect the percentage positive among those samples that were tested.
