# Supplementary material for: Impact of the addition of azithromycin to antimalarials used for seasonal malaria chemoprevention on antimicrobial resistance of Streptococcus pneumoniae
Source: Trop Med Int Health. 2019 Nov 13;24(12):1442–54. doi: 10.1111/tmi.13321 (PMC7687265; doi:10.1111/tmi.13321)
Supplement: Supplementary file 4 — Figure S4. Results of disc diffusion assays for testing for resistance to erythromycin and its comparison to resistance to azithromycin in isolates obtained during three annual pre‐and post‐intervention surveys and 1 year after the last post‐intervention survey was done in Mali. [file TMI-24-1442-s004.pdf]

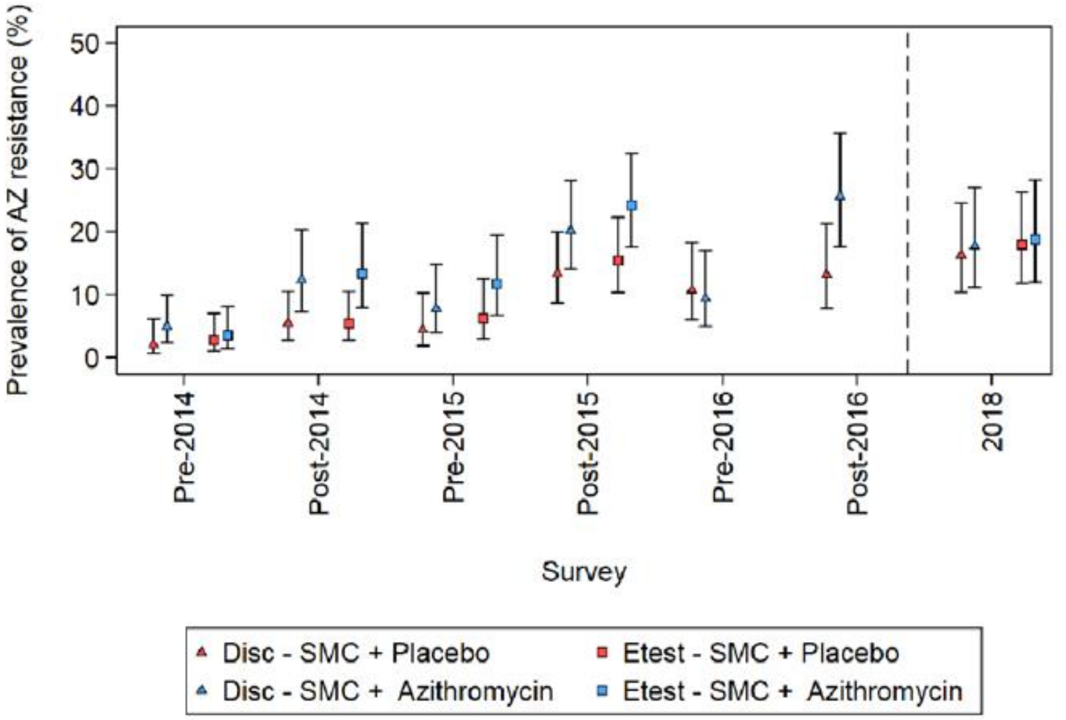

* In 2016 in Burkina Faso, the Azithromycin E-test was used only to confirm samples positive by the Azithromycin disc assay, not to test all samples. Consequently, prevalence by E-test is not comparable to prevalence by Disc Assay, and data points for E-tests are not shown in this figure.
