# Supplementary material for: Impact of the addition of azithromycin to antimalarials used for seasonal malaria chemoprevention on antimicrobial resistance of Streptococcus pneumoniae
Source: Trop Med Int Health. 2019 Nov 13;24(12):1442–54. doi: 10.1111/tmi.13321 (PMC7687265; doi:10.1111/tmi.13321)
Supplement: Supplementary file 5 [file TMI-24-1442-s005.docx]

**THE IMPACT OF THE ADDITION OF AZITHOMYCIN TO THE ANTIMALARIALS USED FOR**

**SEASONAL MALARIA CHEMOPREVENTION ON ANTIMICROBIAL RESISTANCE OF**

***STREPTOCOCCUS PNEUMONIAE***

**Supplementary Materials**

**1. Laboratory methods ................................................................................p. 2**

**2. Investigation of unexpected laboratory findings ......................................p. 3**

**3. Supplementary figures .............................................................................p. 5**

**Laboratory Methods**

***Isolation of Streptococcus pneumoniae and determination of antimicrobial resistance***

Following storage of cryotubes containing skimmed milk-tryptone-glucose-glycerol medium (STGG) inoculated with a nasopharyngeal swab at -80^O^C, 200µl of STGG medium were removed and seeded in Todd Hewitt enrichment medium with 0.5% yeast extract plus 1mL rabbit serum (THY) and incubated for 6 hours in a humid atmosphere containing 5% CO_2_ at 37 ^O^C. After 18 to 24 hours of incubation, 10μl of THY were spread on a gentamicin blood agar plate and incubated for 18-24 hours at 37 ^O^C in 5% CO_2_. Plates were then examined for streptococcal-like alpha-hemolytic colonies. The alpha hemolytic colonies were sub-cultured 24 hours later on a blood agar medium. Pneumococcal identification was based on colony morphology and conventional characterization methods (optochin sensitivity and biliary solubility tests) [1].

The Kirby-Bauer disc diffusion method was used to test pneumococcal isolates for sensitivity to azithromycin (15ug), penicillin (oxacillin 1ug), norfloxacine (10ug) and vancomycin (30ug) according to the recommendations of Clinical and Laboratory Standards Institute (CLSI)[[2](#_ENREF_20)]. Azithromycin and penicillin resistance were confirmed by Epsilometer-test (E-test) with strips of azithromycin and penicillin respectively (Oxoid Ltd). The minimum inhibitory concentration (MICs) were determined. The ceftriaxone E-test was also performed to monitor resistance to this antibiotic. Isolates could not be tested against all antibiotics on all occasions because of problems over the supply of reagents but assays for azithromycin and erythromycin were conducted during each survey using standardised methods.

References.

1. [da Gloria Carvalho M](https://www.ncbi.nlm.nih.gov/pubmed/?term=da%20Gloria%20Carvalho%20M%5BAuthor%5D&cauthor=true&cauthor_uid=20220175), [Pimenta FC](https://www.ncbi.nlm.nih.gov/pubmed/?term=Pimenta%20FC%5BAuthor%5D&cauthor=true&cauthor_uid=20220175), [Jackson D](https://www.ncbi.nlm.nih.gov/pubmed/?term=Jackson%20D%5BAuthor%5D&cauthor=true&cauthor_uid=20220175), et al. Revisiting pneumococcal carriage by use of broth enrichment and PCR techniques for enhanced detection of carriage and serotypes. [J Clin Microbiol](https://www.ncbi.nlm.nih.gov/pubmed?term=%22Journal+of+clinical+microbiology%22%5BJour%5D+AND+48%5Bvolume%5D+AND+1611%5Bpage%5D+AND+2010%5Bpdat%5D&cmd=detailssearch) **2010**; 48: 1611-8.
2. Clinical and laboratory Standards Institute. Performance standards for antimicrobial disc susceptibility tests: approved standard, 26^th^ ed. N1006. 2014

**Retesting of samples giving unexpected results**

Two sets of analyses gave unexpected results, firstly the very high isolation rate of *S. pneumoniae* obtained during the final survey conducted in Mali one year after the last intervention and secondly the very high rate of azithromycin resistance noted in the survey conducted in Mali at the end of the third year of azithromycin administration. To ensure that these unexpected findings did not result from a laboratory error, an attempt was made to re-isolate pneumococci from the vials of inoculated STGG medium that had been stored at -80 ^O^C for one year after collection of the sample.

*Pneumococcal re-isolation rate*

Thirty (50) vials of STGG medium which had originally yielded an isolate of *S. pneumoniae* and 20 vials that were originally culture negative were randomly selected from the full batch of samples available from the 2018 final survey conducted in Mali and stored at – 80 ^O^C until tested. The results are shown in Table S1.

**Table S1.** Results of initial testing and retesting for isolation of *S. pneumoniae* from samples collected in Mali during the survey conducted one year after the last intervention.

|  | Retesting result | |  |
| --- | --- | --- | --- |
| Initial result | *S. pneumoniae* | No growth | Total |
| *S. pneumoniae* | 28 | 2 | 30 |
| No growth | 1 | 19 | 20 |
| Total | 29 | 21 | 50 |

The result of retesting matched the original result in 94.0% of cases (Cohen’s Kappa statistic for inter-observer agreement 87.6%).

*Prevalence of azithromycin resistance*

An attempt was also made to isolate *S. pneumoniae* from samples obtained during the post-2016 survey conducted in Mali (i.e. shortly after the final treatment with azithromycin had been given) which showed a very high prevalence of resistance to AZ, particularly among children who had received AZ. All samples from the SMC + AZ group (37 resistant, 17 sensitive), as well as the 25 positive samples from the SMC + placebo group, were retested for resistance to AZ using the disc diffusion method. The results are shown in Table S2.

**Table S2.** Results of initial testing and retesting for AZ resistance of samples obtained during the last survey conducted in Mali.

|  | Retesting result | |  |
| --- | --- | --- | --- |
| Initial result | Azithromycin resistant | Azithromycin sensitive | Total |
| Azithromycin resistant | 50 | 12 | 62 |
| Azithromycin sensitive | 3 | 14 | 17 |
| Total | 53 | 26 | 79 |

Overall, the result of retesting matched the original result in 81.0% of cases (Cohen’s Kappa statistic for inter-observer agreement, 52.8%). Among the 37 resistant samples from the SMC + AZ group that were retested, 29 (78.4%) were resistant upon re-test. Among the 25 resistant samples from the SMC + placebo that were retested, 21 (84.0%) were resistant upon re-testing.

Based on the confirmatory results obtained on retesting the isolates from the surveys that gave unexpected results, the initial findings have been used in the analyses presented in the main paper.

**SUPPLEMENTARY FIGURES**

**Figure S1**. Schematic showing timing of surveys for pneumococcal sampling in relation to study interventions and the malaria transmission seasons

**Figure S2.** Results of resistance to azithromycin by age obtained during three annual pre-and post-intervention surveys and one year after the last post-intervention survey was done in Burkina Faso (a) and Mali(b).

**Figure S3.** Results of disc diffusion assays for testing for resistance to erythromycin and its comparison to resistance to azithromycin in isolates obtained during three annual pre-and post-intervention surveys and one year after the last post-intervention survey was done in Burkina Faso.

**Figure S4**. Results of disc diffusion assays for testing for resistance to erythromycin and its comparison to resistance to azithromycin in isolates obtained during three annual pre-and post-intervention surveys and one year after the last post-intervention survey was done in Mali.
